# Health status deterioration in subjects with mild to moderate airflow obstruction, a six years observational study

**DOI:** 10.1186/s12931-019-1061-7

**Published:** 2019-05-18

**Authors:** Fernanda Machado Rodrigues, Heleen Demeyer, Matthias Loeckx, Miek Hornikx, Hans Van Remoortel, Wim Janssens, Thierry Troosters

**Affiliations:** 1 Department of Rehabilitation Sciences, KU Leuven - University of Leuven, Leuven, Belgium; 2 Department of Respiratory Diseases, University Hospitals Leuven, Leuven, Belgium; 3 Department of Physiotherapy, LUNEX International University of Health, Exercise and Sports, Differdange, Luxembourg; 4 Department of Cardiovascular Sciences, University Hospitals Leuven, Leuven, Belgium; 5 Centre for Evidence-Based Practice, Belgian Red Cross-Flanders, Mechelen, Belgium; 6 Department of Chronic Diseases, Metabolism and Ageing (CHROMETA), University Hospital Leuven, KU Leuven, Leuven, Belgium

**Keywords:** Health status, Airflow obstruction, Chronic obstructive pulmonary disease, Aging, Longitudinal studies

## Abstract

**Background:**

Patients with COPD need to cope with a disabling disease, which leads to health status impairment.

**Aim:**

To investigate the long term change of health status in subjects with mild to moderate airflow obstruction and to compare this to subjects without airflow obstruction, with and without a smoking history. Second, to investigate the factors potentially associated to rapid health status decline in our total cohort.

**Methods:**

Two hundred and one subjects were included. Generic [Short form 36 health survey (SF36) and EuroQol - 5 dimensions (EQ-5D)] and disease specific [Clinical COPD questionnaire (CCQ) and COPD Assessment Test (CAT)] health status questionnaires were regularly repeated over a six years period. Other functional outcomes comprised measures of lung function, physical fitness, physical activity and emotional state.

**Results:**

On average, health status decline did not differ between groups with the exception of the EQ-5D index, which deteriorated faster in subjects with airflow obstruction compared to the never smoking control group [− 0.018(0.008) versus 0.00006(0.003), *p* = 0.03]. Subjects presenting at least one exacerbation had faster rate of deterioration measured with CAT [0.91(0.21) versus − 0.26(0.25), *p* < 0.01]. Characteristics of the fast declining group were older age, worse lung function, physical fitness, physical activity and disease specific baseline health status. Subjects with airflow obstruction had a 2.5 (95% CI 1.36–4.71) higher risk of presenting fast overall health status decline. Fast overall decline was associated with the presence of acute exacerbation(s) (44% of the subjects with exacerbation(s) versus 17% of subjects without exacerbation, *p* = 0.03). Changes in fat free mass, functional exercise capacity and in symptoms of anxiety and depression correlated weakly to changes in health status measured with all questionnaires.

**Conclusion:**

Subjects with mild airflow obstruction present a significant deterioration of health status, which is generally not much faster compared to smoking and never smoking controls. Subjects with fast decline in overall health status are older and more likely to have airflow obstruction, acute respiratory exacerbation(s), reduced physical fitness, physical activity and impaired COPD specific health status at baseline.

**Trial registration:**

NCT01314807 - retrospectively registered on March 2011.

**Electronic supplementary material:**

The online version of this article (10.1186/s12931-019-1061-7) contains supplementary material, which is available to authorized users.

## Background

Health is defined by the World Health Organization (WHO) as “a state of complete physical, mental and social well-being, and not merely the absence of disease or infirmity” [1]. Health status is one of the recommended targets in the management of Chronic Obstructive Pulmonary Disease (COPD). Patients suffering from COPD present persistent airflow limitation, and systemic consequences, such as muscle dysfunction, impaired exercise tolerance, symptoms of dyspnea and fatigue and impaired health status [2].

Despite its overwhelming impact, COPD is still widely underdiagnosed [3–5]. Especially in its mild or early stage, COPD often remains under the diagnostic radar [3]. Undiagnosed subjects already have a decreased health status compared to healthy controls. When diagnosed, patients present an even worse outcome [4]. Therefore, it is likely that (the perception of) a deteriorated health status is a trigger for patients to seek for medical attention. Indeed, it has already been shown that patients with COPD are more likely to be referred to a respiratory specialist if they experience poor health status [6].

The deterioration of health status over time has previously been described by large and robust studies [7–12]. These studies evaluated patients with moderate to severe stages of COPD. While cross-sectional studies showed that health status was impaired in subjects with mild or undiagnosed airflow obstruction [13, 14], longitudinal change of health status in the early stages of COPD has not been described yet. This information would help to understand how the impaired health status of clinical patients with COPD is developed over time.

The present study aimed to 1) investigate the change in health status of subjects with mild to moderate airflow obstruction over six years as compared to two control groups without airflow obstruction, with and without a significant smoking history, 2) to compare the baseline and change of health status between subjects with or without at least one acute exacerbation, 3) to characterize the subjects who have a faster deterioration in overall health status during the follow up and, finally, 4) to investigate possible associations between decline in health status and in functional outcomes in all participants.

We hypothesized that subjects with mild to moderate airflow obstruction would present faster deterioration in health status compared to the control groups, particularly when experiencing acute exacerbation(s) during follow up.

## Methods

### Design and subjects

Information regarding the study design, subjects recruitment and inclusion criteria has been described elsewhere [15, 16]. Briefly, this study is part of the Rainbow study, a six years prospective, case-control, observational study, which aimed to investigate the prevalence, severity and incidence of systemic consequences in subjects with newly detected mild and moderate airflow obstruction. Three groups were included: 1) subjects with mild to moderate airflow obstruction (‘airflow obstruction’, reference group), 2) (ex-) smokers with a significant smoking history but without airflow obstruction (‘smoking controls’), and 3) never or ex-smokers with a marginal (< 10 packyears) smoking history (‘never smoking controls’). The rainbow study was approved by the ethics committee of the University Hospital Leuven (B3220096387) and subjects were included after signing a written informed consent term. The study was retrospectively registered in the ClinicalTrials.gov (NCT01314807) on March 2011.

### Measurements

#### Health status

The main outcome of this analysis was health status, assessed through self-administered patient reported outcomes (PROs). Generic [Short form 36 health survey (SF36) and Generic EuroQol - 5 dimensions (EQ-5D)], as well as disease specific [Clinical COPD questionnaire (CCQ) and COPD Assessment Test (CAT)] PROs were collected. The smoking groups with and without airflow obstruction responded to the SF36 and the EQ-5D at baseline, one, two, three and six years, while the CCQ was completed at every year until the end of the six years follow up. The never smoking control group was only evaluated at baseline, three and six years and in all visits all the PROs were collected. The CAT was introduced in the study 16 months after its start.

The SF36 is a generic instrument widely used. It has eight domains that can be summarized in two summary scores: Physical component summary (PCS) and Mental component summary (MCS) ranging from 0 (worse) to 100 (best) [17]. The EQ-5D questionnaire is a generic instrument containing two sections: the utility index (index) and the visual analog scale (VAS). The index is calculated from five items (mobility, self-care, usual activities, pain/discomfort and anxiety/depression) ranging from 1 to 3. For the Dutch speaking Belgian population, the final score ranges from − 0.158 to 1, with higher scores indicating better health status and death being scored as zero [18]. The VAS ranges from 0 (worst) to 100 (best), in which subjects indicate how they rate their general health status. The CCQ is a disease-specific questionnaire consisting of three domains (symptoms, functional and mental) and a total score, which ranges from zero to six and higher scores indicate worse health related quality of life [19]. The CAT is a disease-specific instrument evaluating the impact of the disease on the patient’s well-being and daily life. The total score ranges from zero (best) to 40 (worst) [20].

#### Other outcomes

At inclusion and at every follow up visit, a comprehensive interview was performed to assess the clinical history and smoking status. The use of long acting beta agonists, long acting anticholinergics or inhaled corticosteroids was considered maintenance respiratory pharmacotherapy. Acute exacerbations were defined as a variation on respiratory symptoms which required a change in medication or hospitalization in the group with airway obstruction [2]. Functional outcomes were lung function, physical fitness, physical activity (PA) and emotional state:

Lung function was measured according to European Respiratory Society recommendations [21, 22], retrieving values of forced expiratory volume in one second (FEV_1_), functional residual capacity (FRC) and diffusion capacity for carbon monoxide (TL,_CO_). Values were compared to those predicted by Quanjer et al. [23] and were used as natural units for decline.

The following physical fitness outcomes were assessed: 1) body weight and height, resulting in body mass index (BMI); 2) fat free mass (FFM), assessed by dual energy X-ray absorptiometry (DXA) scan (QDR 4500A, Discovey scanners, Hologic, Inc., Bedford, MA, USA) and expressed as percentage of body weight and as FFM index (FFM/ height*height); 3) handgrip force, measured by the Jamar hydraulic hand dynamometer (model J00105, Sammons Preston Inc., Bolingbrook, Illinois), taking the highest of three reproducible isometric contractions. Subjects were seated with arms unsupported and elbow flexed at 90° along de body. Results were compared to the normative values proposed by Mathiowetz et al. [24]; 4) Quadriceps force, via maximal voluntary isometric contraction, with the computerized dynamometer (Biodex system 4 pro – Enraf Nonius; Delft, the Nederlands) [25]. Reference values were calculated as previously described [26]; 5) Functional exercise capacity, as the distance during the six minutes walking test (6MWD) [27]; 6) Maximal exercise capacity, by a maximal incremental cycling test [16], taking peak oxygen uptake (VO_2_peak) and the oxygen uptake efficiency slope (OUES) as the main outcomes. Reference values of VO_2_peak were those reported by Jones et al. [28].

Physical activity (PA) was assessed by an accelerometer (Sensewear Pro 2 Armband Bodymedia, Pittsburgh, PA) worn on the upper right arm during waking hours for seven consecutive days. Valid PA measures were those which contained at least four days with data from at least eight hours per day, between 07:00 AM and 20:00 PM [29]. The number of steps per day and the total time spent in moderate to vigorous PA (above 3 METS - MVPA) were retrieved as variables of interest.

Finally, symptoms of anxiety and depression were assessed by the Hospital Anxiety and Depression scale (HADS) to have information on the emotional status of the subjects [30]. Sum scores for each domain range from zero (best) to 21 (worse). The score threshold of 8 points is suggested as possible case of depression or anxiety [31].

#### Statistical analysis

Data handling and statistical analysis were performed with SAS 9.4 (SAS Institute Inc., Cary, North Carolina, USA). Comparison of continuous data from baseline characteristics were performed by ANOVA or the non-parametric equivalent (Kruskal-Wallis). Frequency of gender, continuous smoking and group distribution, as categorical data, was compared with the chi-square test. Post hoc tests were performed considering the Bonferroni correction for multiple comparisons.

First, to investigate differences in the yearly change in health status among groups, a mixed model was built (PROC mixed) for each of the main outcomes of interest (i.e. SF-36, EQ-5D, CCQ and CAT). Group (class variable with airflow obstruction group as reference), time (continuous variable) and group x time interaction effect (main interest) were inserted in the model. The intercept (initial potency) and slopes (degradation rate), were indicated as random effects. For CAT, we only compared the airflow obstruction group to the smoking control group because the follow up time in the never smoking group was only 2.48 years (compared to the average of 3.95 and 4.1 years follow up from smoking control and airflow obstruction groups). Next, we stratified the analysis for having at least one acute exacerbation (≥1 vs 0 events as class variable) during follow-up among those subjects with airflow obstruction, retrieving the interaction effect. Age was included as covariate in the mixed models to verify any potential interference in the main results.

Second, the individual rate of yearly change in health status was calculated by a simple regression analysis (PROC autoreg). This estimate (slope of the regression) was obtained when at least two measurements during follow up were available. These slopes were used as a sensitivity analysis confirming the mixed model results. Furthermore, the following exploratory analyses were performed, based on the regression slopes:

(I) Characterization of subjects presenting fast decline in overall health status. This was defined based on an arbitrary sum score based on the rate of deterioration (slopes) quartiles of SF36 PCS, SF36 MCS, EQ-5D VAS and CCQ. These questionnaires cover the aspects of physical, mental, general and disease specific health status. EQ-5D index and CAT were not included because their aspects were already covered by the previous instruments and because the scores calculation of EQ-5D index is country dependent and the implementation of CAT was not done at the start of the study.

For each one of the selected instruments, subjects scored 0 (quartile of the slowest decline) to 3 (quartile of the fastest decline). The quartile scores in each of those 4 instruments were summed for each subject. Those with a sum score higher than 9 were classified as presenting fast decline in overall health status. This cut off was chosen because it is the threshold which covers all statistically significant average declines in those four instruments and due to the break of the linearity of the frequency distribution seen at the inspection of the histogram.

In case of missing values in at least one of the instruments, the sum score was also considered missing. This was overruled in case the category was already stablished and would not change, independently of any potential result of those missing results (2 out of 20 cases). For comparisons (sum scores > 9 versus <=9), the independent T-test or the Mann-Whitney test was applied.

The relative risk of presenting fast decline if being in the airflow obstruction group was investigated by the probability of presenting fast decline if being in the airflow obstruction group divided by the probability of presenting fast decline if being in any of the control groups.

(II) Correlation of changes in health status with changes in functional outcomes (i.e. lung function, physical fitness, physical activity and emotional state) was investigated using the Spearman correlation coefficient.

For visual representation of the deterioration in the outcomes of interest, the average and standard error estimated for each visit by the mixed models were plotted in graphs using GraphPad Prism version 8 (GraphPad Software Inc., San Diego, California, USA).

## Results

### Baseline characteristics

**Fig. 1:**
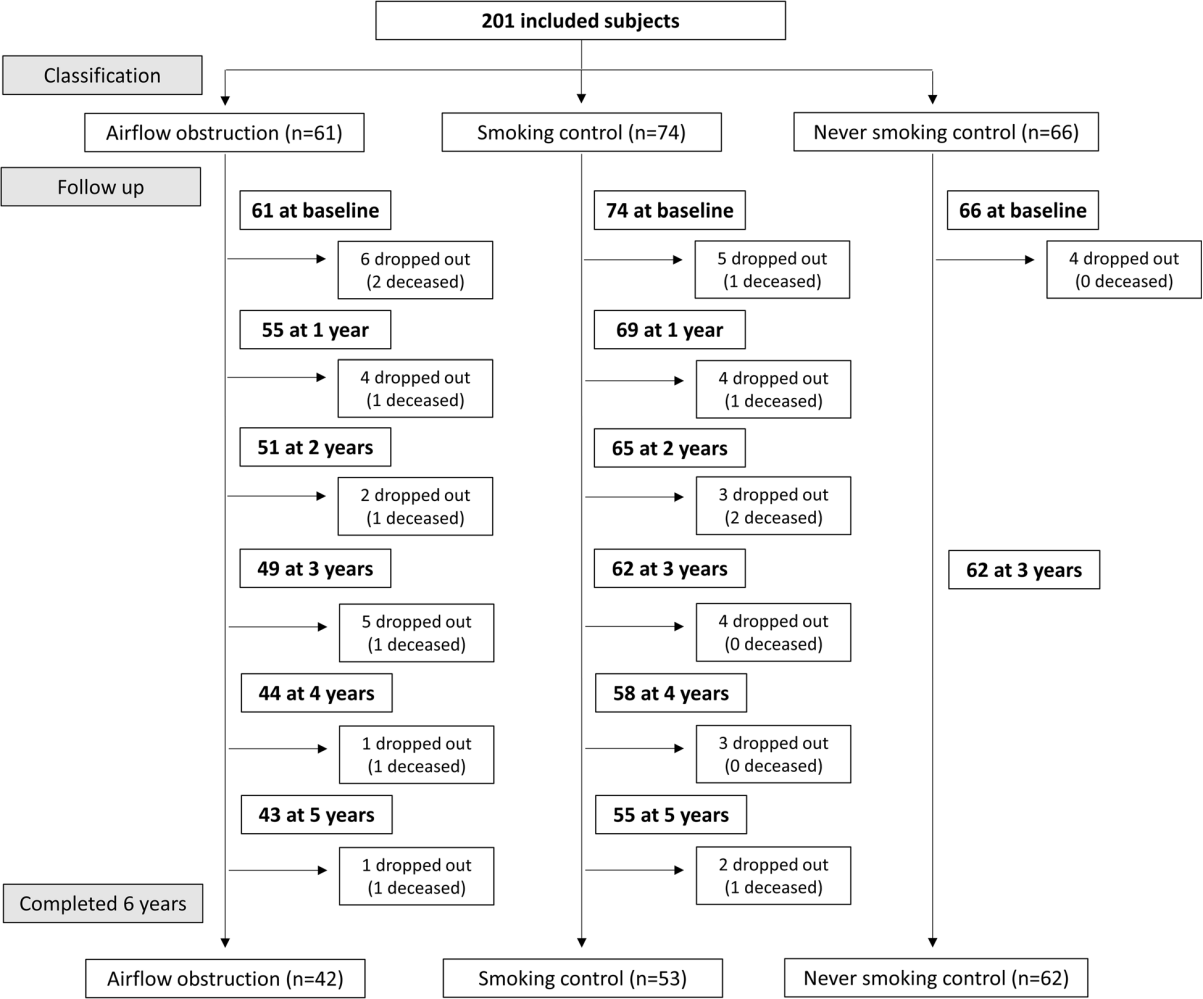
CONSORT type flow chart of inclusions and follow up. Drop outs are indicated immediately after the last attended visit. In case of decease, this is also indicated after the last attended visit by the subject, even if the decease occurred at a later stage. The number of deceases between two visits are included in the number of drop outs and are specified within brackets

From the 201 subjects initially included in the Rainbow cohort, 12 deceased and 32 dropped out of the study along the follow up, resulting in 157 subjects who completed the six years follow up. The time of drop out is depicted at the CONSORT like flow chart of inclusions and follow up (Fig. 1). Drop-out rate was smaller in the never smoking control group than in the smoking control and airflow obstruction groups (6% versus 30 and 31%, *p* < 0.001). Those who dropped out had higher smoking history and presented worse lung function, functional exercise capacity, symptoms of anxiety and health status (Additional file 1: Table S1). Cause of deceases was mainly cancer (lung – 2 cases, mouth, esophagus, brain, blood and bile duct). Other causes were complications after lung volume surgery, heart failure, neurologic condition and suicide. We were not able to retrieve the information for one occurrence.

The characteristics of the subjects included in the study can be found in Table 1. Subjects in the smoking control group were somewhat younger than those with airflow obstruction (*p* = 0.03). Five subjects from the never smoking control group had an irrelevant smoking history (ranging between one to seven packyears, with smoking cessation five to 27 years before entering the study). The frequency distribution of subjects actively smoking during the study period did not differ between the groups of smokers with or without airflow obstruction. Sixty five percent of the airflow obstruction group was composed by subjects with mild obstruction (FEV_1_ ≥ 80% predicted). The remaining 35% was composed by subjects with moderate airflow obstruction (50 ≤ FEV_1_ < 80% predicted).

**Table 1:** Baseline characteristics

	Airflow obstruction (*n* = 61)	Smoking control (*n* = 74)	Never smoking control (*n* = 66)	p
Age (years)	64 ± 7	60 ± 7 ^c^	61 ± 7	0.03
Gender [n (%men)]	45 (74)	45 (61)	36 (55)	0.07
Smoking history (packyears)	48 ± 21	35 ± 20	0.38 ± 1.46^b^	< 0.0001
Active Smoking during study [n (%)]	30 (50)	44 (60)	0 (0) ^cd^	< 0.0001
Lung function				
FEV_1_ (% predicted)	85 ± 16	104 ± 14	116 ± 19^b^	< 0.0001
FRC (% predicted)	129 ± 26^de^	108 ± 17	112 ± 19	< 0.0001
TL,_CO_ (% predicted)	79 ± 17	87 ± 14	96 ± 16^b^	< 0.0001
Physical fitness				
BMI (kg/m^2^)	27 ± 4	27 ± 4	25 ± 3	0.08
FFM (% body weight)	73 ± 7	73 ± 7	73 ± 7	0.92
Handgrip force (% predicted)	99 ± 17	100 ± 18	108 ± 20^cd^	0.01
Quadriceps force (% predicted)	98 ± 22	95 ± 17	111 ± 26^cd^	< 0.0001
6MWD (meter)	587 ± 84	608 ± 69	673 ± 75^cd^	< 0.0001
VO_2_peak (% predicted)	111 ± 28	122 ± 32	129 ± 33^c^	< 0.01
OUES (slope)	2623 ± 628	2606 ± 588	2704 ± 780	0.60
Physical activity				
Steps per day	7814 ± 3786^de^	9532 ± 3759	10,387 ± 3326	< 0.001
MVPA (minutes)	82 ± 66^de^	114 ± 67	115 ± 54	< 0.01
Emotional state				
HADS anxiety (score)	4 [3–7]	4 [1–6]	3 [2–6]	0.16
HADS depression (score)	2 [1–4]	2 [1–4]	1 [0–2]^cd^	< 0.0001
Health status measures				
SF36 PCS (sum score)	76.2 [64.6–81.3]	81.2 [71.5–85.4]	85.8 [78.8–90]^b^	< 0.0001
SF36 MCS (sum score)	79.5 [72.8–85]	82.2 [77.8–87]	86 [82–90.6] ^cd^	< 0.0001
EQ-5D index (score)	0.76 [0.74–1]	0.76 [0.76–1]	1 [0.76–1]	0.05
EQ-5D VAS (score)	75 [70–85]	80 [75–85]	80 [80–90] ^cd^	< 0.01
CCQ (total score)	0.7 [0.4–1.5]	0.4 [0.2–0.7]	0.2 [0.1–0.4]^b^	< 0.0001
CAT (score)^a^	10 [5–14]	8 [4.5–11]	5 [3–8] ^cd^	< 0.0001

Data are expressed as mean ± SD, number (%) or median [interquartile range]. *FEV*_*1*_ forced expiratory volume in one second, *FRC* Functional residual capacity, *TL,*_*CO*_ diffusion capacity for carbon monoxide, *BMI* body mass index, *FFM* fat free mass, *6MWD* six minutes walking distance, *VO*_*2*_*peak* peak oxygen uptake, *OUES* oxygen uptake efficiency slope, *MVPA* time spent in moderate to vigorous physical activity, *HADS* Hospital Anxiety and Depression Scale, *SF36* Short form 36 health survey, *PCS* physical component summary, *MCS* mental component summary, *EQ-5D* Generic EuroQol 5 dimensions, *VAS* visual analog scale, *CCQ* Clinical COPD Questionnaire, *CAT* COPD assessment test (^a^= data from the third year visit as data collection was only initiated 18 months after the start of the study). Missing values: FRC n = 4, TL,_CO_ n = 3, FFM n = 15, handgrip n = 5, quadriceps n = 8, 6MWD n = 5, VO_2_peak and OUES *n* = 10, physical activity *n* = 15, HADS n = 6, SF36 *n* = 5, EQ-5D VAS n = 6, EQ-5D index n = 6, CCQ *n* = 13, CAT *n* = 41. ^b^ = statistical difference among all groups; ^c^ = statistically different from airflow obstruction; ^d^ = statistically different from smoking control; ^e^ = statistically different from never smoking control

As expected, all lung function tests were worse in the group with mild to moderate airflow obstruction compared to both control groups. Measures of BMI and FFM as percentage of body weight were comparable among groups. Handgrip force, quadriceps force, functional and maximal exercise capacity and physical activity were to some extend decreased in subjects with airflow obstruction when compared to the control group(s). Emotional state was slightly worse in subjects with airflow obstruction as they scored more on depressive symptoms than the never smoking control group. Only 12, 7, 1.5% (*p* = 0.07) of subjects had a possible diagnosis of depression in the airflow obstruction, smoking and never smoking control groups, respectively. All the patient reported outcomes (PROs) used to measure health status indicated impairment in the airflow obstruction group compared to the never smoking control (Table 1).

### Change in health status over time

**Fig. 2:**
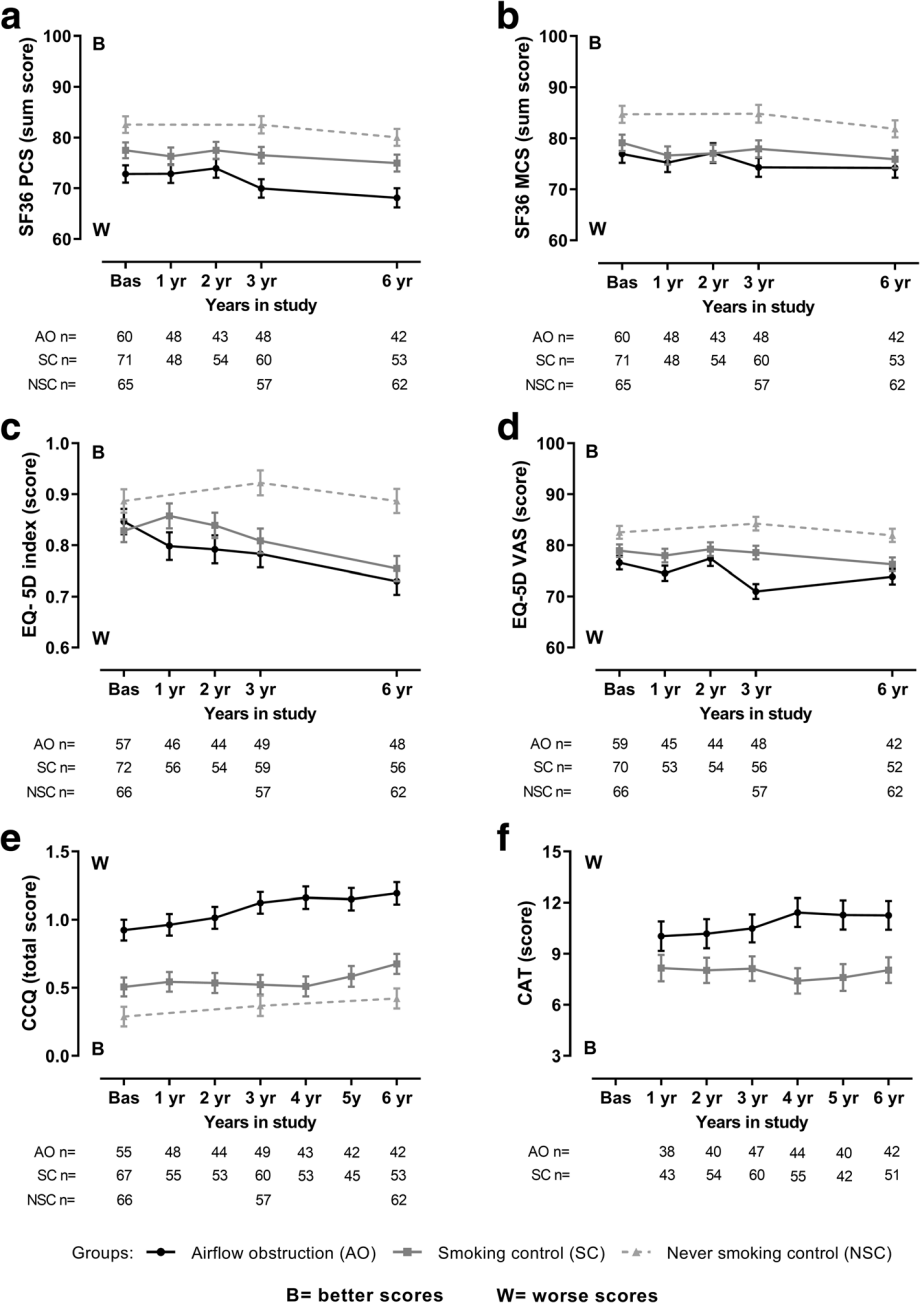
Visual representation of the changes in health status obtained with the mixed model. Data as average and standard error. Panel **a)** Short form 36 health survey physical component summary (SF36 PCS); **b**) Short form 36 health survey mental component summary (SF36 MCS); **c**) Generic EuroQol 5 dimensions utility index (EQ-5D index); **d**) Generic EuroQol 5 dimensions visual analog scale (EQ-5D VAS); **e**) Clinical COPD Questionnaire (CCQ); **f**) COPD assessment test (CAT). The airflow obstruction group (AO) is represented by circles and solid black line, the smoking control group (SC) by squares and solid dark grey line and the never smoking control group (NSC) by triangles and dashed light grey line. AO n=, SC n= and NSC n = refer to the number of valid of measurements in the three groups, in each time-point, for each instrument

Across the entire cohort, health status measured by all PROs, with exception of CAT, significantly decreased over time (Table 2). The deterioration in health status measured by EQ-5D index was faster in the group with airflow obstruction than in the never smoking control group (interaction effect *p* = 0.03) (Table 2). Numerically, SF36 PCS, EQ-5D VAS, CCQ and CAT scores had a faster decline in the airflow obstruction group but this was not statistically significant compared to the controls. The introduction of age as a covariate in the mixed models did not change the overall data (Additional file 2: Table S2). The results obtained with the regression analysis confirmed the findings from the mixed models (Additional file 3: Table S3). A visual representation of the changes in health status measured with the different instruments is depicted in Fig. 2.

**Table 2:** Estimated yearly rate of change in health status

	All	Group		
	Yearly change	Classification	Yearly change	*p* value Interaction effect
	P value			
SF36 PCS (sum score)	−0.560 (0.162)	Airflow obstruction (ref)	−0.983(0.355)*	–
(generic)	p < 0.001	Smoking control	−0.444(0.248)	0.18
		Never smoking control	−0.434(0.253)	0.18
SF36 MCS (sum score)	−0.440 (0.160)	Airflow obstruction (ref)	−0.481(0.362)	–
(generic)	p < 0.01	Smoking control	−0.486(0.288)	0.93
		Never smoking control	−0.483(0.182)*	0.92
EQ-5D index (score)	−0.010 (0.003)	Airflow obstruction (ref)	−0.018(0.008)*	–
(generic)	p < 0.01	Smoking control	−0.013(0.006)*	0.59
		Never smoking control	0.00006(0.003)	0.03
EQ-5D VAS (score)	−0.325 (0.129)	Airflow obstruction (ref)	−0.597(0.285)*	–
(generic)	*p* = 0.01	Smoking control	−0.461(0.247)	0.64
		Never smoking control	−0.095(0.157)	0.13
CCQ (total score)	0.031 (0.007)	Airflow obstruction (ref)	0.054(0.019)*	–
(disease specific)	*p* < 0.0001	Smoking control	0.021(0.009)*	0.05
		Never smoking control	0.023(0.008)*	0.08
CAT (score)	0.073 (0.109)	Airflow obstruction (ref)	0.285(0.187)	–
(disease specific)	*p* = 0.50	Smoking control	−0.103(0.124)	0.09

Data are expressed as mean (standard error). SF36 = Short form 36 health survey, PCS = physical component summary, MCS = mental component summary, EQ-5D = Generic EuroQol 5 dimensions, VAS = visual analog scale, CCQ = Clinical COPD Questionnaire, CAT = COPD assessment test. * indicates a statistical significant change (*p* < 0.05)

### Acute exacerbations

The comparison of baseline and yearly change of health status between subjects with or without at least one acute exacerbation during the follow up can be found in Table 3. From the 61 subjects with airflow obstruction, 25 subjects experienced at least one exacerbation (36 events in total). The baseline health status from those who experienced exacerbation(s) during the follow up did not differ significantly from those without events. The disease specific instruments (CCQ and CAT) captured a numerically worse health status at baseline in the group with at least one exacerbation. The deterioration of health status measured by the CAT was faster in the group of subjects with exacerbation(s). SF36 PCS and SF36 MCS declined numerically faster in subjects with events compared to those free of events, but this difference did not reach statistically significance.

**Table 3:** Comparison of baseline and yearly change of health status between subjects with or without at least one acute exacerbation during the follow up, among those with airflow obstruction

	Exacerbation(s) during follow up	No exacerbation during follow up	p
	(*n* = 25)	(*n* = 36)	
Baseline			
SF36 PCS (sum score)	76.4 [65.1–82]	76 [64.6–81.3]	0.92
SF36 MCS (sum score)	79.5 [72.5–84.3]	80.1 [74.0–85.8]	0.79
EQ-5D index (score)	0.76 [0.74–1]	0.77 [0.76–1.00]	0.70
EQ-5D VAS (score)	75 [70–80]	80 [70–85]	0.48
CCQ (total score)	1.2 [0.3–1.5]	0.70 [0.40–1.00]	0.22
CAT (score)^a^	10.5 [6–17.5]	8 [5–13]	0.11
Yearly change			
SF36 PCS (sum score)	−1.51 (0.47)	−0.50 (0.53)	0.16
SF36 MCS (sum score)	−1.22 (0.54)	0.03 (0.45)	0.16
EQ-5D index (score)	−0.022 (0.010)	−0.015 (0.012)	0.66
EQ-5D VAS (score)	−0.82 (0.47)	− 0.40 (0.35)	0.48
CCQ (total score)	0.068 (0.025)	0.042 (0.028)	0.63
CAT (score)	0.91 (0.21)	−0.26 (0.25)	< 0.01

Data are expressed as median [interquartile range] or average (SE). Baseline data were compared with a Mann–Whitney test, yearly change with a mixed model. SF36 = Short form 36 health survey, PCS = physical component summary, MCS = mental component summary, EQ-5D = Generic EuroQol 5 dimensions, VAS = visual analog scale, CCQ = Clinical COPD Questionnaire, CAT = COPD assessment test (^a^= data from the third year visit). Missing values: Had acute exacerbation(s) – EQ-5D index and CAT n = 1, CCQ n = 3, in both baseline and yearly change. Did not have acute exacerbation – Baseline: SF36 *n* = 1, EQ-5D index and CCQ *n* = 3, EQ-5D VAS n = 2, CAT *n* = 13. Yearly change: SF36 and EQ-5D index n = 7, EQ-5D VAS *n* = 8, CCQ *n* = 9, CAT *n* = 12

### Exploratory findings

#### Fast decline in health status

One hundred eighty three subjects could be classified regarding the decline in overall health status. Table 4 displays the rate of decline in functional outcomes in the groups with fast and slower decline in health status. Compared to the slow decline, the fast decline group had an accelerated rate of decline in FFM as percentage of body weight, functional exercise capacity and symptoms of anxiety and depression. The Additional file 4: Table S4 presents the baseline characteristics of both groups. Subjects with fast decline in overall health status were older, had worse lung function, lower functional exercise capacity (6MWD), oxygen uptake efficiency (OUES) and physical activity (PA) at baseline compared to the group with a slower decline. The emotional state did not differ between groups. Health status, measured by the CCQ was impaired at baseline in subjects with fast decline. Thirty percent of the subjects with airflow obstruction presented a fast decline in health status, while this percentage was lower in the smoking and never smoking control groups, with respectively 13 and 10% of subjects in the fast decline group. Therefore, the relative risk of being in the fast decline group is 2.5 (95% CI 1.36–4.71) fold increased in the airflow obstruction group compared to that in the control groups combined. The occurrence of at least one acute exacerbation in the airflow obstruction group predisposed to fast decline in overall health status. Forty four percent of people experiencing exacerbation(s) were classified in the fast decline group compared to 17% of those without events (*p* = 0.03).

**Table 4:** Comparison of decline in functional outcomes and in health status between groups with fast and slower decline in health status

	Fast decline	Slower decline	p
	(*n* = 31)	(*n* = 152)	
Lung function			
∆ FEV_1_ (liter)	−0.04 [− 0.05 – − 0.02]	-0.02 [− 0.05–0.00]	0.09
∆ FRC (liter)	0.027 [− 0.047–0.086]	0.009 [−0.036–0.064]	0.74
∆ TL,_CO_ (ml/min/kPa)	− 0.047 [− 0.220 – − 0.010]	−0.067 [− 0.173–0.010]	0.74
Physical fitness			
∆ BMI (kg/m^2^)	0.06 [− 0.10–0.26]	0.09 [− 0.06–0.24]	0.88
∆ FFM (% body weight)	−0.49 [− 0.77 – − 0.17]	−0.22 [− 0.45–0.03]	0.02
∆ Handgrip (kg)	−0.49 [− 1.29 – − 0.11]	−0.33 [− 0.83–0.21]	0.08
∆ Quadriceps force (Nm)	−3.83 [− 6.07 – − 1.92]	−3.20 [− 6.56 – − 0.57]	0.61
∆ 6MWD (meter)	−7.27 [− 13.02 – − 1.00]	− 2.12 [− 6.94–3.26]	< 0.01
∆ VO_2_peak (l/min)	−0.06 [− 0.10 – − 0.02]	−0.075 [− 0.13 – − 0.03]	0.50
∆ OUES (slope)	− 45 [− 103–7]	− 57 [− 116 – − 2]	0.58
Physical activity			
∆ Steps/day	− 222 [− 591 – − 66]	− 268 [− 525–92]	0.72
∆ MVPA (minutes)	−2.72 [− 6.50–3.92]	−1.38 [− 7.86–4.21]	0.89
Emotional state			
∆ HADS anxiety (score)	0.32 [−0.03–0.56]	−0.08 [− 0.33–0.13]	< 0.0001
∆ HADS depression (score)	0.28 [0.00–0.81]	0.00 [− 0.17–0.16]	< 0.001
Health status			
∆ SF36 PCS (sum score)	−3.02 [− 5.13 – − 2.13]	0.02 [− 0.89–0.59]	< 0.0001
∆ SF36 MCS (sum score)	−2.38 [− 4.88 – − 0.95]	0.14 [− 0.47–0.76]	< 0.0001
∆ EQ-5D VAS (score)	−2.49 [− 4.57 – − 1.18]	0 [− 0.73–0.84]	< 0.0001
∆ CCQ (total score)	0.100 [0.050–0.177]	0.007 [− 0.020–0.039]	< 0.0001

Data are expressed as median [interquartile range]. FEV_1_ = forced expiratory volume in one second, FRC = Functional residual capacity, TL_CO_ = diffusion capacity for carbon monoxide, BMI = body mass index, FFM = fat free mass, 6MWD = six minutes walking distance, MVPA = time spent in moderate to vigorous physical activity, HADS = Hospital Anxiety and Depression Scale. Missing values: Fast decline – FEV_1_, FRC, TL,_CO_, handgrip force, 6MWD and HADS *n* = 1, FFM index *n* = 8, quadriceps force *n* = 2, VO_2_peak and OUES *n* = 3. Slower decline – FFM index *n* = 18, 6MWD, EQ-5D, CCQ n = 1, VO_2_peak, OUES and CCQ *n* = 7, physical activity *n* = 11

#### Correlation

The results of the correlation between the yearly rate of decline in health status and in functional outcomes are shown in the Additional file 5: Table S5. The deterioration in functional exercise capacity, in fat free mass as percentage of body weight and in symptoms of anxiety and depression was consistently correlated to the decline in health status measured by SF36 PCS, SF36 MCS, EQ-5D VAS and CCQ. The strength of this correlation was, at best, moderate. The decline in EQ-5D VAS correlated weakly with the decline in FEV_1_ and in handgrip force.

## Discussion

### Summary of findings

It has been previously reported that patients with mild to moderate airflow obstruction present health status impairment [13, 14]. To the best of our knowledge, this is the first study with a prospective and controlled design to show the six years longitudinal change of health status in early stages of airflow obstruction compared to smoking and non-smoking control groups. We showed that the decline in health status measured by the EQ-5D index is faster in subjects with mild to moderate airflow obstruction compared to relevant control populations (Table 2 and Fig. 2). Other instruments (SF36 PCS, EQ-5D VAS, CCQ and CAT) also presented the same pattern of somewhat more deterioration in the airflow obstruction group, but without statistically significance (Table 2 and Fig. 2). Second, we showed that subjects with airflow obstruction experiencing at least one acute exacerbation during follow up had a trend for worse COPD specific health status at baseline and had a faster deterioration measured by the CAT score (Table 3).

Our exploratory analyses showed that our cohort with mild to moderate airflow obstruction is at increased risk of having a fast deterioration of overall health status, based on an arbitrary sum score including all instruments (Additional file 4: Table S4 and main text of results). Those subjects with faster decline in overall health status presented a faster decline in functional outcomes such as fat free mass, functional exercise capacity and emotional state (Table 4). The occurrence of acute exacerbation(s) in the airflow obstruction group was also associated to faster decline in overall health status (main text of results). Finally, in all participants, the decline in health status was consistently but weakly correlated to changes in fat free mass, functional exercise capacity and emotional state (Additional file 5: Table S5).

Impaired health status is well documented in patients with moderate to very severe COPD [2]. Therefore, we hypothesized that, by following up subjects with newly diagnosed and early stages of airflow obstruction, we could detected a faster rate of decline in health status compared to control groups. In line with this hypothesis, most instruments used to measure health status (both generic and disease specific) in our study showed a slightly faster deterioration of health status in the airflow obstruction group. Only EQ-5D index presented a statistically significant faster decline in the airflow obstruction compared to the never smoking control group. The fact that this instrument could detect a significant difference is likely due to its ability to provide a value to decease (score 0) [18]. Indeed, mortality was slightly higher in the airflow obstruction group and was mainly caused by smoking related conditions.

### Health status deterioration in the present cohort compared to others

Previous literature reported on the disease specific St George’s Respiratory Questionnaire (SGRQ) to assess the deterioration in health status of patients with more advanced COPD. The SGRQ was not included in the present study. The calculation of the time to reach a clinically meaningful deterioration in health status enables the comparison of results among studies using different instruments. Our cohort of mild to moderate airflow obstruction presented a slower rate of deterioration than the previously reported on more severe COPD. In the Uplift [9] and Eclipse [12] studies, a clinically meaningful deterioration occurs after four years (0.99 and 1.3 points per year in the SGRQ score, respectively). In our cohort it occurs after seven years in the disease specific questionnaires (CCQ and CAT), which was substantially longer.

Exacerbations have a significant effect on health status deterioration in patients with more severe COPD [7, 32, 33]. The impact of the acute exacerbations in milder disease was not studied so far. We found that subjects experiencing at least one exacerbation during the follow up had a numerically worse baseline health status measured by the COPD specific instruments (CCQ and CAT). The average scores, however, were still lower than those reported in typical patients with COPD [34, 35]. Although the between group differences in our study were not statistically different, their magnitude exceeded the minimal clinically important difference previously proposed for these instruments [36, 37]. Miravitlles et al. [33] also found impaired baseline health status in patients with moderate to very severe COPD experiencing frequent exacerbations (i.e. ≥2 per year). In our study, all instruments presented a numerically higher rate of decline in the group which experienced at least one exacerbation during the follow-up. Only CAT showed a statistically faster deterioration in health status compared to the group without events. The lack of consistent statistically significant findings in this comparison is probably due to the relatively low sample size, the low number of exacerbations and the stratification based on at least one acute exacerbation during the entire follow up.

### Exploratory findings

#### Airflow obstruction and infrequent exacerbations are related to fast deterioration in overall health status

We isolated a subgroup of subjects (17% of the whole sample) with consistent fast decline in health status across the different instruments aiming to minimize the bias of using a single instrument. Subjects with airflow obstruction had a 2.5 fold increased risk to be in this group of fast decline in overall health status. While the general difference in decline of health status between subjects with airflow obstruction and controls seems to be modest, this increased risk deserves the attention of clinicians who see these patients in primary care. The occurrence of exacerbation(s) in the group of subjects with airflow obstruction was also related to fast deterioration in overall health status. These findings reinforce the need to closely follow up these subjects in order to prevent exacerbations and, consecutively, the accelerated deterioration in health status.

#### Baseline characteristics of subjects with fast deterioration in overall health status

Our findings confirmed data from the TORCH study [7] by showing that a rapid loss of health status was related to older age. Subjects singled out as having fast decline in overall health status in our analysis presented worse lung function, physical fitness outcomes and had lower PA than those presenting slower decline. This could potentially be associated with frailty developed at older age. The Eclipse study also found a functional limitation measured with the timed up and go test (TUG) to be associated with a clinical important deterioration in health status of patients with COPD [38]. These results corroborate our findings, although our cohort with airflow obstruction presented more preserved lung function and physical fitness outcomes.

Additionally, in the Eclipse study [38], baseline symptoms of depression and worsening in dyspnea also were predictors of deterioration in health status. This was not confirmed in the present cohort. However, scores for depressive symptoms were generally low at baseline. The disease specific CCQ, which comprises symptoms in its assessment, was worse at baseline in our subjects with fast decline.

Although subjects in the fast decline in overall health status did not present faster deterioration in PA, they did present lower levels of PA at baseline compared to the slower overall decline group. Physical inactivity is known to be related to the development of several chronic diseases and to poor prognosis [39]. Therefore, it is not surprising that subjects presenting fast decline in health status were less active than those with slower decline. A large epidemiologic study in China also showed that PA is associated with better psychological well-being, lower likelihood of having chronic diseases and disabilities and a slower cognitive impairment over time [40].

The group with fast deterioration in overall health status was not exclusively composed by subjects with airflow obstruction. The other characteristics above mentioned (higher age, worse functional exercise capacity and oxygen uptake efficiency and less physical activity) could be risk factors for cardiovascular and metabolic comorbidities. These risk factors might have contributed to the faster deterioration in health status of control subjects [41, 42].

Surprisingly, the quadriceps force, as percentage of the predicted, was higher in the fast decline group. This is counter intuitive, but probably reflects the average older age and lower body weight, both impacting on the predicted quadriceps force in this group. The values of quadriceps force, corrected by body weight, tended to be lower in the group with fast decline, although this difference was not statistically significant.

### Deterioration in functional outcomes of subjects with fast deterioration in overall health status

Deterioration in FFM (% body weight), functional exercise capacity and symptoms of anxiety and depression was faster in the fast health status group compared to the slower decline. While the criteria chosen to classify fast deterioration might be arbitrary, the results from the correlation analysis between changes in health status and in functional outcomes in all subjects were reassuring, although correlations were generally weak. Changes in symptoms of anxiety and depression, also measured by a patient reported outcome, consistently correlated with changes in health status. Previous studies also indicated a weak correlation between function and health status [8, 10, 43], underscoring the importance of measuring patients’ reported health status. Interestingly, the change in fat free mass as percentage of body weight and functional exercise capacity were also consistently (but moderately) related to decline in health status. This provides further validity to this outcome as an integrated measure of overall health in elderly with or without airflow obstruction.

### Strengths and limitations

Previous studies have mainly focused on moderate to very severe stages of COPD in either observational [10, 11, 38] or interventional [7–9] designs, mostly without including a healthy aging control group. In the present study, the included subjects were diagnosed with mild to moderate airflow obstruction as a result of a screening spirometry in a population based study from our center rather than following a clinical diagnosis. This allowed the investigation of the longitudinal changes from an early onset of the disease. Furthermore, two relevant control groups, without airflow obstruction, were included for comparison.

The previous literature made use of the St George’s Respiratory Questionnaire (SGRQ), a disease specific instrument widely used in research, but with limited use in clinical practice, due to its extensiveness. This study, in contrast, used a robust set of reliable, generic and disease specific instruments to measure health status in our cohorts. Furthermore, the instruments included in our study allowed the investigation of health status in subjects who did not necessarily presented important symptoms during daily activities.

However, the present study also has some limitations. Subjects who dropped out during the follow up (including deceases) were mainly included in the airflow obstruction (42% of drop outs) and smoking control (49% of drop outs) groups. They had more smoking exposure, worse lung function, functional capacity, higher symptoms of anxiety and worse health status. This might have led to a selection bias of the present findings, with a probable underestimation of the differences between airflow obstruction group and controls. Indeed, the subjects from the fast decline group presented worse baseline functional outcomes than those from the slower decline group. However, we tried to limit this selection bias by maximally using the available data.

This study lacks the power to detect statistically significant differences, especially in the stratification and exploratory analyses. Nevertheless, the authors feel that the study provides a unique insight in the trajectory of health status in (former) smokers from the population with and without mild to moderate airflow obstruction and may provide data to set up fully powered studies to investigate the impact of exacerbations in patients in the early stages of developing clinical COPD.

### Future perspectives

Despite this comprehensive design, the onset of the disabling health status was, unfortunately, not captured in this study. This would probably demand an epidemiological study with much larger proportions in terms of sample size and/or follow up duration, starting the inclusion at earlier ages and before the onset of airflow obstruction. This may not be feasible at a population level. Therefore, tackling subjects at higher risk of rapid deterioration and investigating the effects of early intervention in this subgroup would be of more relevance for future studies. Our results indicated that subjects with airflow obstruction, acute exacerbation(s), worse diffusion capacity, hyperinflation, exercise capacity and physical activity are predisposed for overall fast health status deterioration.

The instruments selected to measure health status in the current study are designed to assess general and specific disease conditions. Nevertheless, they seemed to lack the capability to detect subtle changes at the onset of the disease. The development of a patient reported outcome specifically geared to people with mild disease might be worthwhile to detect subtle changes.

## Conclusion

Subjects with mild airflow obstruction present a significant deterioration of health status, which is generally not much faster compared to smoking and never smoking controls. Subjects with fast decline in overall health status are older and more likely to have airflow obstruction, acute respiratory exacerbation(s), reduced physical fitness, physical activity and impaired COPD specific health status at baseline.

## Additional files

Additional file 1: **Table S1.** Characteristics of subjects who completed six years follow up and those who dropped out. (DOCX 14 kb)

Additional file 2: **Table S2.** Estimated yearly rate of change in health status with the addition of age as a covariate in the model. (DOCX 14 kb)

Additional file 3: **Table S3.** Estimate of the yearly change in health status calculated by the regression analysis. (DOCX 12 kb)

Additional file 4: **Table S4.** Comparison of baseline characteristics between groups with fast and slow decline in health status. (DOCX 14 kb)

Additional file 5: **Table S5.** Spearman correlation between changes in health status and changes in functional outcomes. (DOCX 15 kb)

## Abbreviations

6MWD: Distance during the six minutes walking test
BMI: Body mass index
CAT: COPD assessment test
CCQ: Clinical COPD questionnaire
COPD: Chronic obstructive pulmonary disease
DXA: Dual energy X-ray absorptiometry
EQ-5D: EuroQol - 5 dimensions questionnaire
FEV_1_: Forced expiratory volume in one second
FFM: Fat free mass
FRC: Functional residual capacity
HADS: Hospital Anxiety and Depression Scale
MVPA: Moderate to vigorous physical activity
OUES: Oxygen uptake efficiency slope
PA: Physical activity
PRO: Patient reported outcome
SF36 MCS: Short form 36 health survey – mental component summary score
SF36 PCS: Short form 36 health survey – physical component summary score
TL,_CO_: Diffusion capacity for carbon monoxide
VAS: Visual analog scale
VO_2_peak: Peak oxygen uptake
WHO: World health organization
