# Supplementary material for: Health status deterioration in subjects with mild to moderate airflow obstruction, a six years observational study
Source: Respir Res. 2019 May 18;20:93. doi: 10.1186/s12931-019-1061-7 (PMC6525445; doi:10.1186/s12931-019-1061-7)
Supplement: Supplementary file 1 — Table S1. Characteristics of subjects who completed six years follow up and those who dropped out. (DOCX 14 kb) [file 12931_2019_1061_MOESM1_ESM.docx]

Table S1. Characteristics of subjects who completed six years follow up and those who dropped out

|  | Completed follow up  (n=157) | Dropped out  (n=44) | p |
| --- | --- | --- | --- |
| Age (years) | 62±6 | 61±9 | 0.44 |
| Gender [n (%men)] | 99 (63%) | 27 (61%) | 0.84 |
| Smoking history (packyear) | 25±26 | 36±23 | 0.01 |
| Lung function |  |  |  |
| FEV_1_ (liter) | 3.01±0.75 | 2.67±0.80 | <0.01 |
| FEV_1_ (% predicted) | 105±19 | 92±23 | 0.0001 |
| FRC (liter) | 3.71±0.81 | 3.96±0.97 | 0.08 |
| FRC (% predicted) | 114±21 | 122±27 | <0.05 |
| TL,_CO_ (ml/min/kPa) | 7.72±1.99 | 7.25±1.93 | 0.17 |
| TL,_CO_ (% predicted) | 89±17 | 82±17 | 0.02 |
| Physical fitness |  |  |  |
| BMI (kg/m^2^) | 26±4 | 27±4 | 0.43 |
| FFM index (kg/m^2^) | 19±3 | 20±3 | 0.53 |
| Handgrip force (% predicted) | 103±19 | 100±18 | 0.38 |
| Quadriceps force (% predicted) | 103±24 | 96±19 | 0.12 |
| Quadriceps force (Nm/kg) | 2.08±0.47 | 1.99±0.51 | 0.28 |
| 6MWD (meter) | 630±79 | 596±94 | 0.02 |
| VO_2_peak (% predicted) | 123±32 | 113±30 | 0.09 |
| OUES (slope) | 2647±681 | 2627±609 | 0.87 |
| Physical activity |  |  |  |
| Steps per day | 9395±3583 | 8792±4344 | 0.36 |
| MVPA (minutes) | 108±63 | 92±68 | 0.15 |
| Emotional function |  |  |  |
| HADS anxiety (score) | 3 [2-6] | 5 [3-8] | 0.04 |
| HADS depression (score) | 2 [0-3] | 3 [1-5] | 0.05 |
| Health status measures |  |  |  |
| SF36 PCS (sum score) | 82.2 [74.3-86.8] | 75.4 [67.2-82.7] | <0.01 |
| SF36 MCS (sum score) | 83.7 [78.6-88] | 79.3 [72.9-85.2] | <0.01 |
| EQ-5D index (score) | 1 [0.76-1] | 0.76 [0.74-1] | 0.02 |
| EQ-5D VAS (score) | 80 [75-86] | 80 [70-85] | 0.04 |
| CCQ (total score) | 0.3 [0.2-0.6] | 0.6 [0.4-1.3] | <0.001 |
| CAT (score)^ | 6 [4-11] | 10 [6-14] | 0.06 |

Data are expressed as mean±std, number (%) or median [interquartile range]. FEV_1_= forced expiratory volume in one second, FRC= Functional residual capacity, TL_CO_= diffusion capacity for carbon monoxide, BMI= body mass index, FFM= fat free mass, 6MWD= six minutes walking distance, VO_2_peak= peak oxygen uptake, OUES= oxygen uptake efficiency slope, MVPA= time spent in moderate to vigorous physical activity, HADS= Hospital Anxiety and Depression Scale, SF36= Short form 36 health survey, PCS= physical component summary, MCS= mental component summary, EQ-5D= Generic EuroQol 5 dimensions, VAS= visual analog scale, CCQ= Clinical COPD Questionnaire, CAT= COPD assessment test (^data from the third year visit). Missing values: Completed follow up – FRC n= 3, TL,_CO_ n= 2, FFM index n= 12, handgrip force, 6MWD n= 4, VO_2_peak, OUES, HADS, EQ-5D and SF36 n= 5, quadriceps force n= 7, physical activity and CAT n= 13, CCQ n= 8. Dropped out – FRC, TL,CO, handgrip force, quadriceps force, 6MWD, HADS, SF36 and EQ-5D n= 1, FFM index n= 3, VO_2_peak, OUES and CCQ n= 5, physical activity n= 2 and CAT n= 29.
