# Supplementary material for: Health status deterioration in subjects with mild to moderate airflow obstruction, a six years observational study
Source: Respir Res. 2019 May 18;20:93. doi: 10.1186/s12931-019-1061-7 (PMC6525445; doi:10.1186/s12931-019-1061-7)
Supplement: Supplementary file 2 — Table S2. Estimated yearly rate of change in health status with the addition of age as a covariate in the model. (DOCX 14 kb) [file 12931_2019_1061_MOESM2_ESM.docx]

Table S2. Estimated yearly rate of change in health status with the addition of age as a covariate in the model.

|  | All |  | Group | | |
| --- | --- | --- | --- | --- | --- |
|  | Yearly change |  | Classification | Yearly change | p value  Interaction effect |
|  | P value |  |  |  |  |
| SF36 PCS (sum score) | -0.559 (0.162) |  | Airflow obstruction (ref) | -0.983(0.355)* | - |
|  | p < 0.001 |  | Smoking control | -0.445(0.248) | 0.18 |
|  |  |  | Never smoking control | -0.433(0.252) | 0.18 |
|  |  |  |  |  |  |
| SF36 MCS (sum score) | -0.439 (0.159) |  | Airflow obstruction (ref) | -0.483(0.362) | - |
|  | p < 0.01 |  | Smoking control | -0.485(0.288) | 0.91 |
|  |  |  | Never smoking control | -0.483(0.183)* | 0.94 |
|  |  |  |  |  |  |
| EQ-5D index (score) | -0.010 (0.003) |  | Airflow obstruction (ref) | -0.018(0.008)* | - |
|  | p < 0.01 |  | Smoking control | -0.013(0.006)* | 0.60 |
|  |  |  | Never smoking control | -0.00005(0.003) | 0.03 |
|  |  |  |  |  |  |
| EQ-5D VAS (score) | -0.324 (0.129) |  | Airflow obstruction (ref) | -0.603(0.285)* | - |
|  | p = 0.01 |  | Smoking control | -0.458(0.246) | 0.64 |
|  |  |  | Never smoking control | -0.096(0.157) | 0.13 |
|  |  |  |  |  |  |
| CCQ (total score) | 0.031 (0.007) |  | Airflow obstruction (ref) | 0.055(0.019)* | - |
|  | p < 0.0001 |  | Smoking control | 0.021(0.009)* | 0.05 |
|  |  |  | Never smoking control | 0.023(0.008)* | 0.08 |
|  |  |  |  |  |  |
| CAT (score) | 0.075 (0.109) |  | Airflow obstruction (ref) | 0.174(0.163) | - |
|  | p = 0.49 |  | Smoking control | -0.025(0.154) | 0.09 |

Data are expressed as mean (standard error). SF36= Short form 36 health survey, PCS= physical component summary, MCS= mental component summary, EQ-5D= Generic EuroQol 5 dimensions, VAS= visual analog scale, CCQ= Clinical COPD Questionnaire, CAT= COPD assessment test. * indicates a statistical significant change (p<0.05).
