# Supplementary material for: Health status deterioration in subjects with mild to moderate airflow obstruction, a six years observational study
Source: Respir Res. 2019 May 18;20:93. doi: 10.1186/s12931-019-1061-7 (PMC6525445; doi:10.1186/s12931-019-1061-7)
Supplement: Supplementary file 3 — Table S3. Estimate of the yearly change in health status calculated by the regression analysis. (DOCX 12 kb) [file 12931_2019_1061_MOESM3_ESM.docx]

Table S3. Estimate of the yearly change in health status calculated by the regression analysis.

|  | Airflow obstruction | Smoking control | Never smoking control |
| --- | --- | --- | --- |
|  | Change/year | Change/year | Change/year |
| SF36 PCS (sum score) | -0.94 [-3.34 – 0.85] | -0.16 [-1.54 – 0.41] | -0.11 [-0.87 – 0.35] |
| SF36 MCS (sum score) | -0.25 [-1.31 – 0.62] | -0.0001 [-1.29 – 1.01] | -0.10 [-0.87 – 0.37] |
| EQ-5D index (score) | 0 [-0.04 – 0.008] | 0 [-0.02 – 0] | 0 [0 – 0] |
| EQ-5D VAS (score) | -0.31 [-1.84 – 0.39] | -0.02 [-1.51 – 0.85] | 0 [-0.81 – 0.82] |
| CCQ (total score) | 0.046 [-0.011 – 0.11] | 0.016 [-0.020 – 0.043] | 0.016 [-0.0008 – 0.04] |
| CAT (score) | 0.42 [-0.36 – 1.03] | 0.05 [-0.49 – 0.48] | 0 [-0.99 – 0.68] |

Data are expressed as median [interquartile range]. SF36= Short form 36 health survey, PCS= physical component summary, MCS= mental component summary, EQ-5D= Generic EuroQol 5 dimensions, VAS= visual analog scale, CCQ= Clinical COPD Questionnaire, CAT= COPD assessment test.
