# Supplementary material for: Health status deterioration in subjects with mild to moderate airflow obstruction, a six years observational study
Source: Respir Res. 2019 May 18;20:93. doi: 10.1186/s12931-019-1061-7 (PMC6525445; doi:10.1186/s12931-019-1061-7)
Supplement: Supplementary file 4 — Table S4. Comparison of baseline characteristics between groups with fast and slow decline in health status. (DOCX 14 kb) [file 12931_2019_1061_MOESM4_ESM.docx]

Table S4. Comparison of baseline characteristics between groups with fast and slow decline in health status.

|  | Fast decline | Slow decline | p |
| --- | --- | --- | --- |
|  | (n=31) | (n=152) |  |
| Age (years) | 66±6 | 61±7 | <0.001 |
| Gender [n (%men)] | 16 (52) | 103 (68) | 0.09 |
| Smoking history (packyear) | 32±26 | 26±26 | 0.22 |
| Active Smoking during study [n (%)] | 15 (48) | 52 (34) | 0.13 |
| Group distribution |  |  | 0.01 |
| Airflow obstruction [n (%)] | 16 (30) | 38 (70) |  |
| Smoking control [n (%)] | 9 (13) | 58 (87) |  |
| Never smoking control [n (%)] | 6 (10) | 56 (90) |  |
| Lung function |  |  |  |
| FEV_1_ (liter) | 2.52±0.79 | 3.07±0.74 | <0.001 |
| FEV_1_ (% predicted) | 97±25 | 104±18 | 0.09 |
| FRC (liter) | 3.90±0.99 | 3.76±0.81 | 0.41 |
| FRC (% predicted) | 125±32 | 114±20 | 0.01 |
| TL,_CO_ (ml/min/kPa) | 6.46±1.99 | 7.96±1.91 | 0.0001 |
| TL,_CO_ (% predicted) | 79±19 | 90±16 | <0.01 |
| Physical fitness |  |  |  |
| BMI (kg/m^2^) | 26±4 | 26±4 | 0.29 |
| Body weight (kg) | 72±13 | 78±14 | 0.04 |
| FFM (% body weight) | 73±7 | 74±7 | 0.38 |
| Handgrip (% predicted) | 106±21 | 101±18 | 0.21 |
| Quadriceps force (% predicted) | 112±33 | 100±20 | <0.01 |
| Quadriceps force (Nm/kg) | 1.99±0.45 | 2.10±0.47 | 0.26 |
| 6MWD (meter) | 579±69 | 635±81 | <0.001 |
| VO_2_peak (% predicted) | 118±31 | 122±32 | 0.54 |
| OUES (slope) | 2332±570 | 2731±677 | <0.01 |
| Physical activity |  |  |  |
| Steps/day | 7613±3046 | 9704±3764 | <0.01 |
| MVPA (minutes) | 81±53 | 112±66 | 0.02 |
| Emotional state |  |  |  |
| HADS anxiety (score) | 5 [2-7] | 3 [2-6] | 0.26 |
| HADS depression (score) | 2 [1-4] | 2 [0-3] | 0.07 |
| Health status |  |  |  |
| SF36 PCS (sum score) | 83.2 [69-86.4] | 81.2 [72.7-86.4] | 0.95 |
| SF36 MCS (sum score) | 80.2 [73.3-87.6] | 83.6 [78.4-88] | 0.43 |
| EQ-5D VAS (score) | 80 [75-88] | 80 [75-85] | 0.53 |
| CCQ (total score) | 0.6 [0.3-1.1] | 0.3 [0.2-0.7] | 0.02 |

Data are expressed as mean±std, number (%) or median [interquartile range]. Fast decline was determined as a high sum (>9) of scores based on quartile split rate of decline in SF36 PCS, SF36 MCS, EQ-5D VAS and CCQ. FEV_1_= forced expiratory volume in one second, FRC= Functional residual capacity, TL,_CO_ = diffusion capacity for carbon monoxide, BMI= body mass index, FFM= fat free mass, 6MWD= six minutes walking distance, MVPA= time spent in moderate to vigorous physical activity, HADS= Hospital Anxiety and Depression Scale. Missing values: Fast decline – FRC, TL_CO_, 6MWD and HADS n= 1, FFM index n= 6, handgrip force, VO_2_peak and OUES n= 2, quadriceps force n= 3. Slow decline – FRC, handgrip force, 6MWD n= 3, TL,CO n= 2, FFM n= 7, quadriceps force n= 5, VO_2_peak and OUES n= 6, physical activity n= 14.
