# Supplementary material for: Health status deterioration in subjects with mild to moderate airflow obstruction, a six years observational study
Source: Respir Res. 2019 May 18;20:93. doi: 10.1186/s12931-019-1061-7 (PMC6525445; doi:10.1186/s12931-019-1061-7)
Supplement: Supplementary file 5 — Table S5. Spearman correlation between changes in health status and changes in functional outcomes. (DOCX 15 kb) [file 12931_2019_1061_MOESM5_ESM.docx]

Table S5. Spearman correlation between changes in health status and changes in functional outcomes.

|  | ∆ SF36 PCS | ∆ SF36 MCS | ∆ EQ-5D VAS | ∆ CCQ |
| --- | --- | --- | --- | --- |
| Lung function |  |  |  |  |
| ∆ FEV_1_ (liter) | r= 0.06, p= 0.39, n= 183 | r= 0.04, p= 0.62, n= 183 | **r= 0.18, p= 0.01, n= 181** | r= -0.12, p= 0.12, n= 181 |
| ∆ FRC (liter) | r= 0.13, p= 0.08, n= 183 | r= -0.05, p= 0.52, n= 183 | r= -0.03, p= 0.64, n= 181 | r= -0.07, p= 0.34, n= 181 |
| ∆ TL,_CO_ (ml/min/kPa) | r= -0.04, p= 0.57, n= 183 | r= -0.08, p= 0.30, n= 183 | r= 0.07, p= 0.35, n= 181 | r= 0.003, p= 0.98, n= 181 |
| Frailty measures |  |  |  |  |
| ∆ BMI (kg/m^2^) | r= -0.06, p= 0.40, n= 184 | r= 0.05, p= 0.47, n= 184 | r= -0.08, p= 0.26, n= 182 | r= 0.12, p= 0.11, n= 182 |
| ∆ FFM (% body weight) | **r= 0.25, p< 0.01, n= 157** | **r= 0.17, p= 0.03, n= 157** | **r= 0.24, p< 0.01, n= 157** | **r= -0.22, p< 0.01, n= 157** |
| ∆ Handgrip force (kg) | r= 0.04, p= 0.54, n= 183 | r= 0.07, p= 0.31, n= 183 | **r= 0.15, p= 0.04, n= 181** | r= -0.10, p= 0.17, n= 181 |
| Physical fitness |  |  |  |  |
| ∆ Quadriceps force (Nm) | r= 0.06, p= 0.39, n= 182 | r= 0.007, p= 0.92, n= 182 | r= 0.04, p= 0.61, n= 180 | r= -0.01, p= 0.88, n= 180 |
| ∆ Quadriceps force (Nm/kg) | r= 0.09, p= 0.23, n= 182 | r= -0.02, p= 0.77, n= 182 | r= 0.05, p= 0.48, n= 180 | r= -0.04, p= 0.52, n= 180 |
| ∆ 6MWD (meter) | **r= 0.36, p< 0.0001, n= 181** | **r= 0.23, p< 0.01, n= 181** | **r= 0.23, p< 0.01, n= 180** | **r= -0.27, p< 0.001, n= 180** |
| ∆ VO_2_peak (l/min) | r= 0.09, p= 0.22, n= 174 | r= -0.002, p= 0.97, n= 174 | r= 0.05, p= 0.51, n= 172 | r= -0.05, p= 0.49, n= 172 |
| OUES | r= 0.03, p= 0.72, n= 174 | r= -0.05, p= 0.54, n= 174 | r= 0.04, p= 0.55, n= 172 | r= 0.03, p= 0.67, n= 172 |
| Physical activity |  |  |  |  |
| ∆ steps/day | r= 0.15, p= 0.05, n= 172 | r= 0.11, p= 0.16, n= 172 | r= 0.10, p= 0.18, n= 171 | r= -0.002, p= 0.98, n= 172 |
| ∆ MVPA (minutes) | r= 0.09, p= 0.23, n= 172 | r= 0.06, p= 0.43, n=172 | r= 0.03, p= 0.69, n= 171 | r= -0.03, p= 0.69, n= 172 |
| Emotional state |  |  |  |  |
| ∆ HADS anxiety (score) | **r= -0.29, p< 0.001, n= 182** | **r= -0.30, p< 0.0001, n= 182** | **r= -0.25, p< 0.001, n= 181** | **r= 0.23, p< 0.1, n= 181** |
| ∆ HADS depression (score) | **r= -0.25, p< 0.001, n= 182** | **r= -0.45, p< 0.001, n= 182** | **r= -0.23, p< 0.01, n= 181** | **r= 0.24, p< 0.01, n= 181** |

SF36= Short form 36 health survey, PCS= physical component summary, MCS= mental component summary, EQ – VAS= Generic EuroQol visual analog scale, CCQ= Clinical COPD Questionnaire, CAT= COPD assessment test. FEV_1_= forced expiratory volume in one second, FRC= Functional residual capacity, TL,_CO_= diffusion capacity for carbon monoxide, BMI= body mass index, FFM= fat free mass, 6MWD= six minutes walking distance, MVPA= time spent in moderate to vigorous physical activity, HADS= Hospital Anxiety and Depression Scale. Statistical significant correlations are highlighted in bold.
